# Healthcare-associated infections during the coronavirus disease 2019 (COVID-19) pandemic and the modulating effect of centralized surveillance

**DOI:** 10.1017/ash.2023.139

**Published:** 2023-04-11

**Authors:** Graham M. Snyder, Suzanne Wagester, Patricia L. Harris, Abby L. Valek, Jacob C. Hodges, Andrew L. Bilderback, Fazrina Kader, Colleen A. Tanner, Amy P. Metzger, Susan E. DiNucci, Bonnie V. Colaianne, Ashley Chung, Rachel L. Zapf, Paula L. Kip, Tamra E. Minnier

**Affiliations:** 1 Department of Infection Prevention and Control, UPMC Presbyterian/Shadyside, Pittsburgh, Pennsylvania; 2 Division of Infectious Diseases, University of Pittsburgh School of Medicine, Pittsburgh, Pennsylvania; 3 Wolff Center, UPMC, Pittsburgh, Pennsylvania; 4 Quality and Risk Management, UPMC Passavant, McCandless, Pennsylvania

## Abstract

We analyzed efficacy of a centralized surveillance infection prevention (CSIP) program in a healthcare system on healthcare-associated infection (HAI) rates amid the coronavirus disease 2019 (COVID-19) pandemic. HAI rates were variable in CSIP and non-CSIP facilities. Central-line–associated bloodstream infection (CLABSI), *C. difficile* infection (CSI), and surgical-site infection (SSI) rates were negatively correlated with COVID-19 intensity in CSIP facilities.

Healthcare-associated infections (HAIs) are a serious complication in patient care that negatively affects patient outcomes and staff safety: ∼4% of hospitalized patients will develop an HAI and ∼11% will die during hospitalization.^1,2^ The coronavirus disease 2019 (COVID-19) pandemic has introduced various challenges in hospital settings, including staffing and capacity management issues. Previous studies have shown that HAI rates have increased during the COVID-19 pandemic.^3^

Typically, HAI transmission in healthcare settings is monitored by a team of local infection preventionists that work onsite at a single facility, in collaboration with other healthcare workers.^4,5^ In our health system, we have developed and implemented a centralized surveillance infection prevention (CSIP) program that includes a team of system-level infection preventionists responsible for HAI surveillance, allowing local infection preventionists to refocus their time on harm reduction efforts.^6^

The aim of this quality improvement work was to analyze HAI rates between sites with CSIP program surveillance and sites without CSIP program surveillance. We also sought to determine the effect of the CSIP program on the relationship between COVID-19 and HAI rates.

## Methods

### Setting

Observations and data collection for this quality improvement initiative were conducted beginning January 2018 and concluding December 2021 at a 40-hospital academic healthcare system. In this analysis, all facilities implementing CSIP programs during the analysis period were assigned identifiers beginning with C (denoting a CSIP facility) and ending with a number assigned in order of the date of CSIP implementation. In total, 6 facilities implemented CSIP in 2019 (C1–C6), 2 facilities implemented CSIP in 2020 (C7, C8), and 4 facilities implemented CSIP in 2021 (C9–C12).

Any facilities that produced data used for this report that did not implement CSIP during the analysis period were designated “local” facilities and were alphabetically assigned an identifier beginning with “L” and ending with a sequential number.

### Intervention

The CSIP program comprises senior infection preventionists performing HAI surveillance and data analysis for facilities adopting centralized surveillance.^6^

### Outcomes and data sources

We performed 2 analyses estimating the impact of CSIP deployment as an effect modifier of the relationship between the COVID-19 pandemic and HAI rates. The first was a comparison of HAI rates in a pre-CSIP pre–COVID-19 period (January–December 2018) to a period when the CSIP program had been deployed at 6 facilities (and 2 additional facilities during the analysis period) during the COVID-19 pandemic (January–December 2020). Second, we compared COVID-19 surge intensity to the outcome of monthly facility HAI rates during March 2020 through December 2021. COVID-19 surge intensity was defined as the proportion of inpatient beds assigned to COVID-19–contagious patients. Surgeries were not limited in these facilities due to COVID-19. Scatter plots were created comparing COVID-19 surge intensity to each HAI rate for each facility month. In our organization, severe acute respiratory coronavirus virus 2 (SARS-CoV-2)–positive patient admissions were not restricted to specific facilities; care was provided at the appropriate facility for the patient’s needs, independent of COVID-19 status. Because HAI incidence is highest in intensive care units and HAI risk during the COVID-19 pandemic is hypothesized to be related to this level of care, we performed a post-hoc correlation analysis restricted to ICU-level care.^7^

In total, 8 CSIP hospitals and 17 hospitals that have not yet implemented CSIP but were eligible based on their electronic health record system (facilities L1–L13) or implemented CSIP after the 2020 analysis period (facilities C9–C12) were included in this analysis. Periods were chosen to accommodate for CSIP implementation. HAIs were determined using NHSN definitions.

### Statistical analysis methods

The comparison of HAI rates in 2018 and 2020 were descriptive in nature and were calculated by subtracting the HAI per 1,000 patient days (in the case of *C. difficile* infection) or catheter days (for CAUTI and CLABSI) in 2020 from the corresponding HAI rate in 2018. To analyze individual facility monthly HAI rates compared to COVID-19 inpatient care intensity, we calculated a Spearman correlation coefficient.

The project underwent formal review and was granted ethical approval (project no. 1905) as a quality improvement project by the Quality Improvement Review Committee.

## Results

**Fig. 1.**
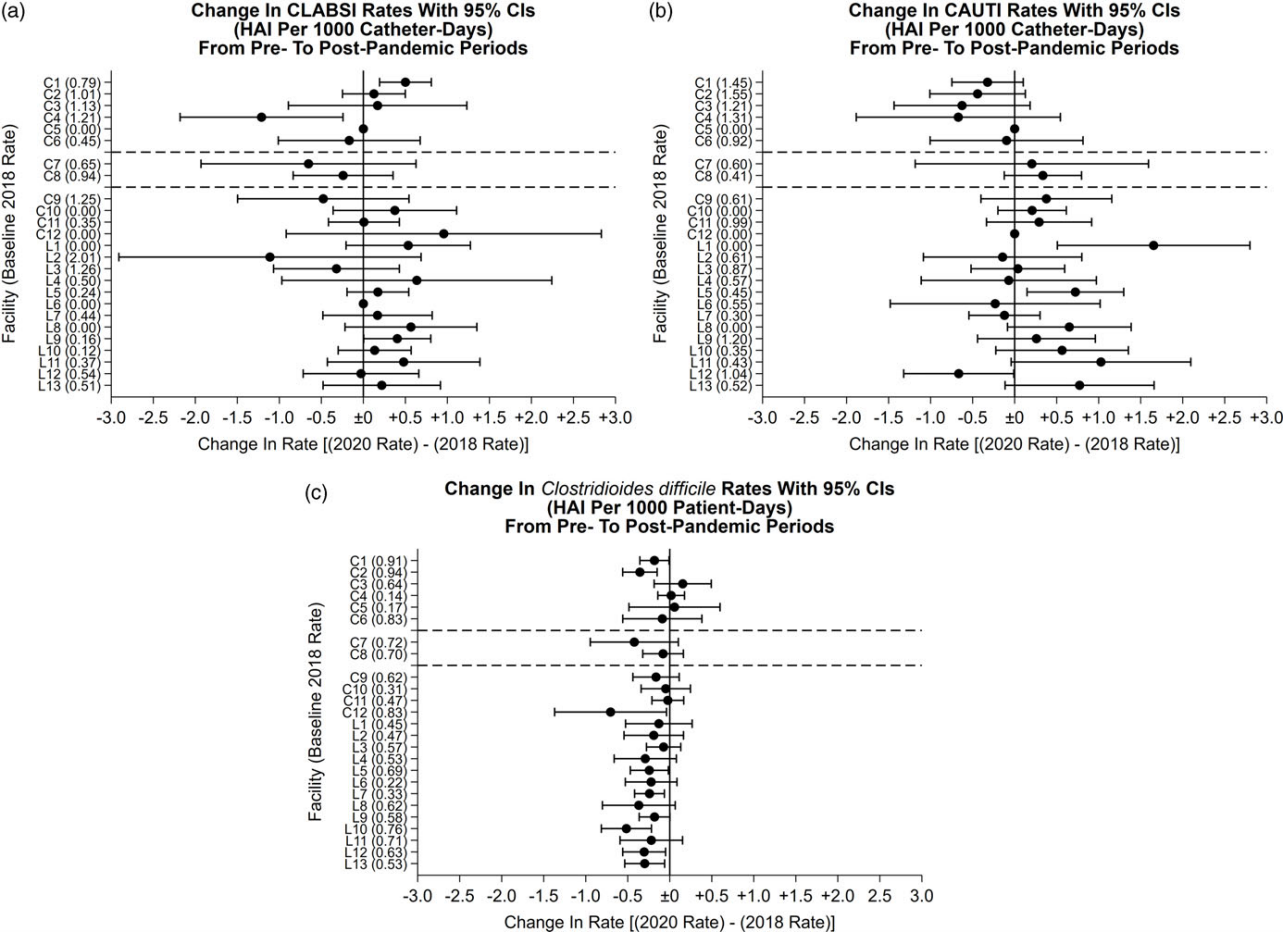
HAI infection rates before and after CSIP. (a) CLABSI rates before and after CSIP implementation and the COVID-19 pandemic. (b) CAUTI rates before and after CSIP implementation and the COVID-19 pandemic. (c) *C. difficile* infection rates before and after CSIP implementation and the COVID-19 pandemic. Facilities above the first dotted line (C1, C2, C3, C4, C5, C6) represent facilities that had CSIP programs in place for the entire post-CSIP analysis period (2020). Facilities above the second dotted line (C7, C8) represent facilities that implemented CSIP during the analysis period; thus, the data from these facilities are a mix of CSIP and non-CSIP data. Facilities below the second dotted line (C9, C10, C11, C12, L1, L2, L3, L4, L5, L6, L7, L8, L9, L10, L11, L12, L13) represent facilities that did not have a CSIP program implemented during the post-CSIP analysis period. Note. CSIP, centralized surveillance infection prevention; HAI, hospital-associated infection; CLABSI, central-line–associated bloodstream infection; CAUTI, catheter-associated urinary tract infection.

We compared CLABSI, CAUTI, and *C. difficile* infection rates in 25 facilities in our system: 6 fully participating in the CSIP program in 2018 and 2020, 2 that implemented CSIP during the 2020 analysis period, and 7 that are eligible for future CSIP program implementation (Fig. 1a–c). When comparing 2 time points in 2018 and 2020, HAI showed variable levels of change among the 25 hospitals (Supplementary Table S1). Non-CSIP hospitals were more likely than CSIP hospitals to report an increase in CLABSI and CAUTI rates in 2020 compared to 2018. Nearly all hospitals had a similar or decreased *C. difficile* rate in 2020.

**Fig. 2.**
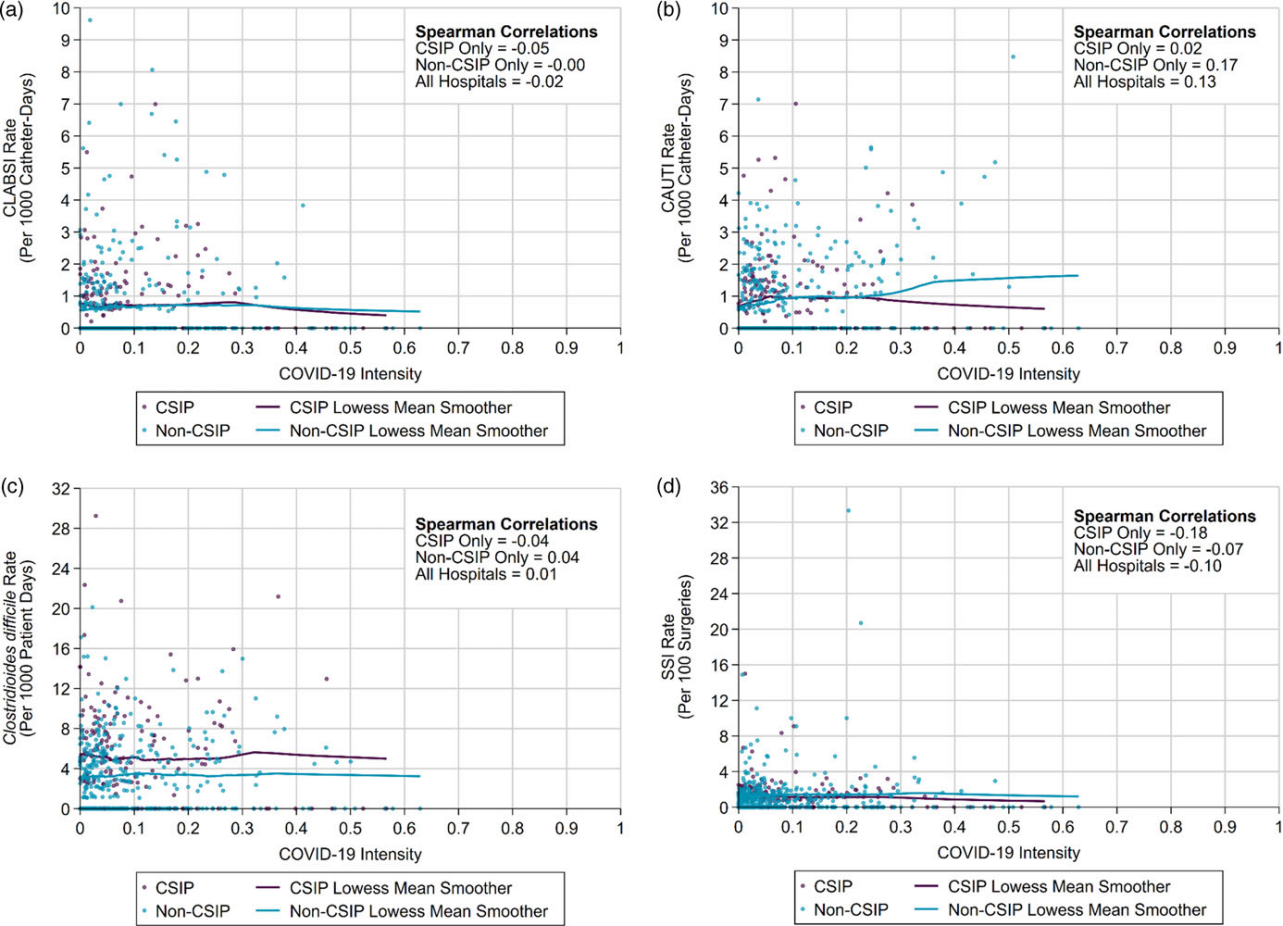
HAI correlated with COVID-19 surge intensity. (a) CLABSI rate correlated with COVID-19 surge intensity. (b) CAUTI rate correlated with COVID-19 surge intensity. (c) CDI rate correlated with COVID-19 surge intensity. (d) SSI rate correlated with COVID-19 surge intensity. Each point is a hospital month of data. Monthly data points are classified as CSIP or non-CSIP based on their predominant surveillance status in that month (for example, facilities switching to the CSIP model would contribute non-CSIP data points until the time of CSIP adoption, after which the facility would contribute to CSIP data points). Note. CSIP, centralized surveillance infection prevention; HAI, hospital-associated infection; CLABSI, central-line–associated bloodstream infection; CAUTI, catheter-associated urinary tract infection; CDI, *C. difficile* infection; SSI, surgical site infection.

COVID-19 intensity was variable over time for all facilities (Supplementary Fig. S1). In the analysis of HAI rates and COVID-19 surge intensity, the correlations in all facilities for each of the 4 HAIs, and the correlations stratified by CSIP and non-CSIP programs all had a Spearman correlation coefficient ρ <0.20 (Fig. 2a–d). Non-CSIP facilities had no correlation between COVID-19 intensity and CLABSI, a weak positive correlation for *C. difficile* infection, and a modest negative correlation for SSI (Fig. 2a, 2c, and 2d). Unlike CSIP hospitals, which had a very weak positive correlation between COVID-19 intensity and CAUTI (ρ = .02), non-CSIP hospitals had a nonmeaningful positive correlation (ρ = .17) (Fig. 2b). The correlation analysis restricted to intensive care unit-level demonstrated no stronger association between HAI rates and COVID-19 intensity. (Supplementary Fig. S2a–c and Supplementary Table S2).

## Discussion

In this quality improvement study characterizing the impacts of CSIP implementation and the COVID-19 pandemic on HAI rates, we identified several major themes. Across our acute-care facilities, there was not a clear pattern of worsening HAI rates because of the COVID-19 pandemic; however, we did observe some evidence that a CSIP-based HAI surveillance program may improve the ability of acute-care facilities to maintain a safe patient environment during major disruptions to providing healthcare. These observations are a substantial contribution to the published experience of centralized surveillance. These results will be important to multiple-facility health systems considering centralized models for HAI surveillance, and our findings provide a suggestion that such a model may improve patient safety in acute-care settings.^8,9^

CSIP programs are ultimately intended to reduce HAI. Our data provide an indeterminate answer regarding whether CSIP program adoption reduces HAI, and these data are confounded by the COVID-19 pandemic. Unlike other studies, however, our analyses showed a mix of both modestly rising and falling rates; consistent with other literature, CAUTI and CLABSI were most adversely impacted.^3,7,10^ Our subpopulation analysis restricted to intensive care unit–level care did not demonstrate that the impact of COVID-19 on HAI rates are attributable to severely ill patients. Other health systems likely also find that the COVID-19 pandemic did not universally negatively affect HAI rates. We have also demonstrated a preliminary signal that CSIP programs allow facilities to maintain HAI prevention progress during times of high patient volume. In the correlation coefficient models we controlled for autoregression and therefore facility trends, but we did not control for HAI reduction initiatives or adherence to infection prevention process measures, which may also have influenced HAI rates.

In this quality improvement project report, we demonstrated that a CSIP program allowed hospitals to maintain HAI prevention progress during COVID-19 surges. Future investigations should continue to characterize ways that CSIP programs can improve infection-related patient safety.

## Supplementary material

For supplementary material accompanying this paper visit http://doi.org/10.1017/ash.2023.139. click here to view supplementary material
