# Supplementary material for: Healthcare-associated infections during the coronavirus disease 2019 (COVID-19) pandemic and the modulating effect of centralized surveillance
Source: Antimicrob Steward Healthc Epidemiol. 2023 Apr 11;3(1):e72. doi: 10.1017/ash.2023.139 (PMC10127231; doi:10.1017/ash.2023.139)
Supplement: Supplementary file 1 [file S2732494X23001390sup001.docx]

**Supplemental Table S1: HAI rates in CSIP and Non-CSIP Hospitals, 2018 to 2020**

| **HAI** | **Change in HAI rate, 2018 to 2020** | | | | |
| --- | --- | --- | --- | --- | --- |
|  | Substantial  decrease | Decrease | Similar/  Unchanged | Increase | Substantial  increase |
| CLABSI | | | | | |
| CSIP hospitals* (N=8) | 2 | 2 | 1 | 2 | 1 |
| Non-CSIP hospitals (N=17) | 1 | 2 | 3 | 7 | 4 |
| CAUTI | | | | | |
| CSIP hospitals | 2 | 3 | 1 | 2 | 0 |
| Non-CSIP hospitals | 1 | 3 | 3 | 4 | 6 |
| *C. difficile* infection | | | | | |
| CSIP hospitals | 0 | 3 | 4 | 1 | 0 |
| Non-CSIP hospitals | 2 | 12 | 3 | 0 | 0 |

Abbreviations: HAI – healthcare-associated infection, CLABSI – central line-associated bloodstream infection, CAUTI – catheter-associated urinary tract infection

*CSIP hospitals were defined as any facility that implemented CSIP during the analysis period (Facilities C1-C8), two facilities transitioned to CSIP during the analysis period (C7 and C8).

Note: Data for this table were drawn from facility-reported HAI rates in the manner described in the text.

**Supplemental Table S2: HAI rates and COVID-19 surge intensity Spearman correlation coefficients**

| **HAI** | **Facilities** | **Full analysis** | **ICU only** | **Delta** |
| --- | --- | --- | --- | --- |
| CLABSI | All facilities | -0.02 | -0.02 | 0 |
|  | CSIP | -0.05 | 0.11 | **+0.16** |
|  | Non-CSIP | -0.00 | -0.03 | -0.03 |
| CAUTI | All facilities | 0.13 | 0.11 | -0.02 |
|  | CSIP | 0.02 | 0.09 | +0.07 |
|  | Non-CSIP | 0.17 | 0.14 | -0.03 |
| *C. difficile* | All facilities | 0.01 | -0.06 | -0.07 |
|  | CSIP | -0.04 | -0.26 | **-0.22** |
|  | Non-CSIP | 0.04 | 0.03 | -0.01 |
| SSI | All facilities | -0.10 | — | N/A |
|  | CSIP | -0.18 | — | N/A |
|  | Non-CSIP | -0.07 | — | N/A |

Abbreviations: HAI – healthcare-associated infection, CLABSI – central line-associated bloodstream infection, CAUTI – catheter-associated urinary tract infection, SSI – surgical site infection

**Supplemental Figure S1: COVID-19 Intensity Over Time, By Facility**





**Supplemental Figure S1 (cont.): COVID-19 Intensity Over Time, By Facility**





Facilities beginning with C denote facilities that implemented CSIP during the analysis period, facilities beginning with L did not implement CSIP during the analysis period.

**Supplemental Figure S2: HAI rates and COVID-19 surge intensity, ICU data only**


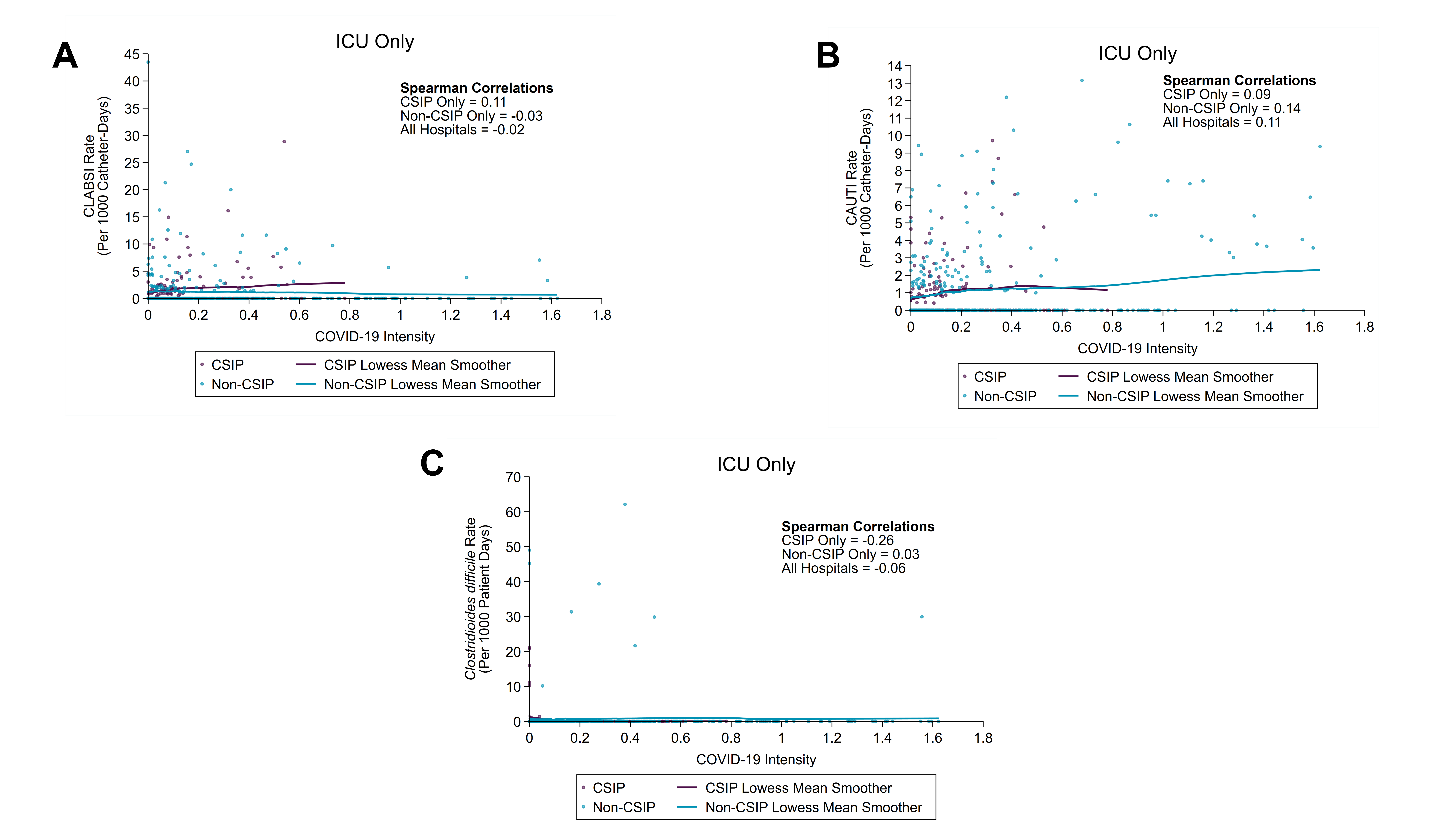


**S2a:** CLABSI rate correlated with COVID-19 surge intensity

**S2b:** CAUTI rate correlated with COVID-19 surge intensity

**S2c:** *C. difficile* infection rate correlated with COVID-19 surge intensity

Legend: Each point is a hospital month of data. Monthly data points are classified as CSIP or non-CSIP based on their predominant surveillance status in that month (for example, facilities switching to the CSIP model would contributed non-CSIP data points until the time of CSIP adoption, after which the facility would contribute to CSIP data points).

Abbreviations: CSIP – centralized surveillance infection prevention, HAI – hospital-associated infection, CLABSI – central line-associated bloodstream infection, CAUTI – catheter-associated urinary tract infection
